# Thrombopoietin may enhance ventilator-induced lung injury

**DOI:** 10.1186/cc9627

**Published:** 2011-03-11

**Authors:** LD Del Sorbo, V Fanelli, G Muraca, EL Martin, L Lutri, A Costamagna, B Assenzio, E Lupia, G Montrucchio, VM Ranieri

**Affiliations:** 1 University of Turin, Italy

## Introduction

Ventilator-induced lung injury is characterized by release of inflammatory mediators and increased vascular permeability resulting in alveolar edema formation. Thrombopoietin (TPO), whose most known function is the stimulation of the proliferation of megakaryocytes, has also shown several proinflammatory effects. Moreover, TPO receptor, c-Mpl, is constitutively expressed on endothelial cells and may modulate the permeability of the endothelium. We investigated the role of TPO in the impairment of the alveolar-capillary membrane resulting in alveolar edema formation during mechanical ventilation.

## Methods

An *ex vivo *model of isolated, ventilated and perfused mouse lung was set up: ventilation was performed for 2 hours with both low-stress pressure (peak inspiratory pressure = 7 cmH_2_O, PEEP = 2 cmH_2_O, RR = 90 beats/minute) and high-stress pressure (peak inspiratory pressure = 20 cmH_2_O, PEEP = 0, RR = 90 beats/minute), in the presence or absence of TPO (1 ng/ml) in the perfusate (2% bovine serum albumin RPMI medium at 1 ml/minute flow rate). At the end of the experiment, lung compliance, assessed through tidal volume, and protein concentration in the bronchoalveolar lavage (BAL) fluid were measured.

## Results

During high-stress ventilation, lung compliance was significantly reduced by the presence of TPO in the perfusate. TPO did not affect compliance during low-stress pressure. BAL fluid protein concentration was increased by the presence of TPO in both pressure setup, but the increase was statistically significant only after high-stress ventilation. See Table 1.

**Table 1:** 

	High-stress MV	High-stress MV + TPO	Low-stress MV	Low-stress MV + TPO
Lung elastance (cmH_2_O/ml)	14.1 ± 1.43	16.5 ± 0.4*	10.2 ± 0.3	9.6 ± 0.4
BAL (μg/g body weight)	19.18 ± 1.69	29.80 ± 3.25*	13.22 ± 1.29	17.71 ± 1.54

Data presented as mean ± SE. *P < 0.05.

## Conclusions

TPO may enhance the permeability of the alveolar-capillary membrane contributing to the mechanisms of ventilator-induced lung injury.

